# Abortion caused by hemolytic disease associated with co-infection by *Anaplasma marginale* and *Babesia* spp. in dairy cattle

**DOI:** 10.1007/s11259-026-11414-x

**Published:** 2026-07-23

**Authors:** Ícaro Guilherme dos Santos, Letícia Perri Almeida Luciano, João Paulo da Silva Cavasani, Marlon Ribeiro, Rafaela Mallmann Bohn, Bernardo Nassau de Souza, João Fabio Soares, Bernardo Coelho de Carvalho, Emílio César Martins Pereira, Marcos de Almeida Souza, Edson Moleta Colodel, Caroline Argenta Pescador

**Affiliations:** 1 Laboratory of Veterinary Pathology, Faculty of Veterinary Medicine, Federal University of Mato Grosso, Av. Fernando Corrêa da Costa, 2367 - Boa Esperança, 78060-900 Cuiabá, MT Brazil; 2 Laboratory of Veterinary Pathology, University of Cuiabá (UNIC), Cuiabá, MT Brazil; 3 Laboratory of Protozoology and Vector-Borne Rickettsiosis, Faculty of Veterinary Medicine, Federal University of Rio Grande do Sul (UFRGS), Porto Alegre, Brazil; 4 Dairy Cattle Sector, Federal University of Mato Grosso (UFMT), Cuiabá, Mato Grosso Brazil

**Keywords:** Fetal loss, Cattle, Rickettsia, Hemolysis

## Abstract

Bovine anaplasmosis and babesiosis are important tick-borne diseases associated with significant economic losses, although fetal infection and abortion are rarely described. This study reports a bovine abortion associated with co-infection by *Anaplasma marginale*, *Babesia bovis* and *Babesia bigemina.* in the Midwestern region of Brazil. Fetal necropsy was performed, followed by cytological, histopathological, immunohistochemical, and molecular analyses. Gross findings included jaundice, splenomegaly, hepatomegaly, and renal discoloration, consistent with severe hemolytic disease. Blood smear evaluation revealed intraerythrocytic inclusions compatible with *(A) marginale* and *Babesia*-like merozoites, and PCR confirmed *(B) bovis* and *B. bigemina*, and *Anaplasma* spp. infection was confirmed by sequencing. Histologically, lesions consisted of erythrophagocytosis, hemosiderosis, hemoglobin casts, and marked intravascular leukocytostasis in multiple organs. Immunohistochemistry demonstrated accumulation of macrophages and T lymphocytes, suggesting activation of a fetal inflammatory response. These findings emphasize the importance of including hemoparasitic infections in the differential diagnosis of bovine abortion and support a role for fetal immune responses in lesion development.

## Introduction

Bovine anaplasmosis and babesiosis are important tick-borne diseases caused by the intraerythrocytic rickettsial bacterium *Anaplasma marginale* and the apicomplexan protozoa *Babesia bigemina* and *Babesia bovis*, respectively, and are widely distributed in cattle-producing regions worldwide (Bock et al., 2004; Marques et al. 2020; MacGregor et al. 2021). In Brazil, transmission occurs primarily through bites of *Rhipicephalus (Boophilus) microplus* ticks, although mechanical transmission of *Anaplasma* spp. by tabanid flies and blood-contaminated instruments has also been reported (Kocan et al. 2010). The epidemiology of these diseases is strongly influenced by climatic conditions, varying across tropical, subtropical, and temperate regions. In subtropical areas, enzootic instability may predispose herds to outbreaks, whereas in tropical regions such as Mato Grosso, where climatic conditions favor continuous tick exposure, infections are typically considered endemic (Almeida et al. 2006; Lucena et al. 2010; Mello et al. 2017; Henker et al. 2020).

Clinically, bovine anaplasmosis and babesiosis are characterized by fever, anemia, weakness, anorexia, dehydration, jaundice, and reduced productivity, and severe cases may progress to death. Infections caused by *B. bovis* may additionally result in neurological signs due to sequestration of parasitized erythrocytes in cerebral capillaries (Bock et al., 2004). Pregnant cows may also develop reproductive disorders, including abortion (Richey and Palmer 1990). Transplacental transmission of *A. marginale* and *Babesia bovis* has been described mainly during the second and third trimesters of gestation (Ribeiro et al., 1995; Henker et al. 2020); however, fetal infection and abortion are considered uncommon outcomes, particularly when concurrent infection with *Babesia* spp. is involved (Correa et al. 1978; Henker et al. 2020).

Although both pathogens are endemic in many cattle-producing areas, reports describing bovine abortion associated with concurrent anaplasmosis and babesiosis, confirmed through integrated pathological and molecular approaches, remain scarce in the literature. In addition, the characterization of fetal lesions associated with these co-infections and their differential diagnoses is still limited, which hampers definitive etiological interpretation in abortion investigations.

Therefore, the present study describes a case of bovine abortion associated with co-infection by *(A) marginale*, *(B) bovis* and *B. bigemina* in the Midwestern region of Brazil, based on gross, histopathological, immunohistochemistry and molecular findings.

## Case presentation

Reproductive failure occurred in a herd of 50 Holstein × Gir crossbred dairy cows belonging to the School Farm of the Federal University of Mato Grosso (UFMT), located in the municipality of Santo Antônio de Leverger, Mato Grosso, Brazil. Animals were maintained on improved pastures (*Brachiaria brizantha* and *Brachiaria humidicola*) with free access to mineral supplementation and water. During the dry season, cows received corn silage twice daily after milking. The herd was routinely vaccinated against *Brucella abortus*, bovine herpesvirus type 1 (BoHV-1) and bovine viral diarrhea virus (BVDV). Artificial insemination (AI) was used as the standard reproductive method, and pregnancy was diagnosed by ultrasonography 30 days after AI.

A 5/8 Girolando bovine fetus at approximately eight months of gestation was submitted to the Veterinary Pathology Laboratory at UFMT for postmortem examination. The period leading up to the abortion was marked by unstable weather and atypical rainfall. The dam was a seven-year-old cow with no additional clinical signs at the time of abortion; however, this was the second abortion recorded for this animal.

**Fig. 1:**
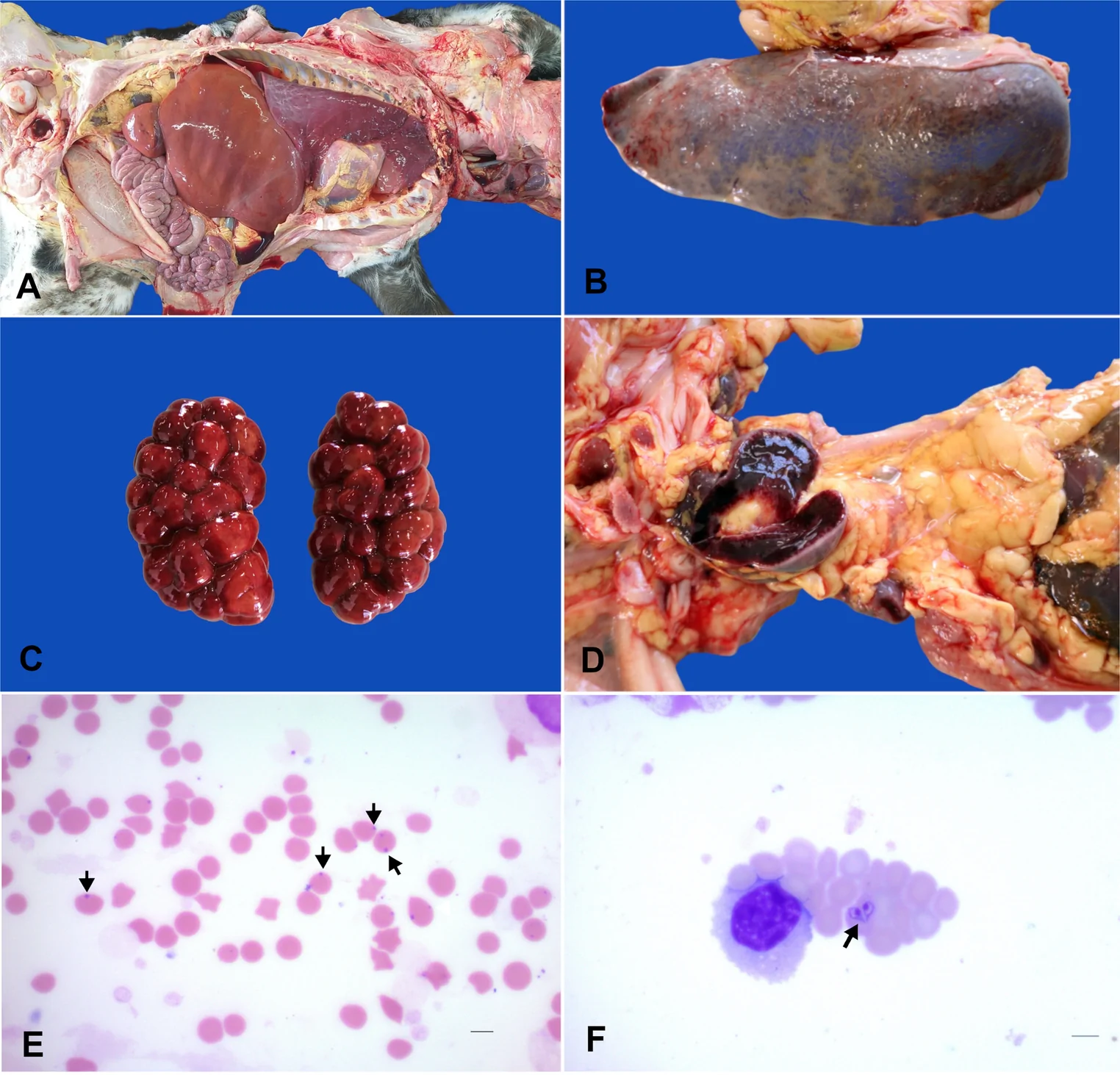
Macroscopic lesions observed during necropsy of a bovine fetus naturally infected with *Anaplasma marginale* and *Babesia bigemina *and cytological analysis. **A**. Upon opening the body cavities, mild generalized icterus and pulmonary collapse were observed. The liver was markedly enlarged and diffusely yellow-orange in color. **B**. Severe splenomegaly. **C**. Kidneys exhibiting diffuse, marked dark red to black discoloration. **D**. Renal lymph node enlarged and diffusely darkened. **E**. Fetal blood smear. Several round intraerythrocytic structures consistent with *A. marginale* (arrows). Obj. 63x, bar 10 μm. **F**. Fetal blood smear. Erythrocytes containing paired pyriform intraerythrocytic organisms consistent with *Babesia* spp. A nearby mononuclear cell (macrophage) is present. Obj. 100x, bar, 5 μm

At necropsy, the fetus measured 74 cm crown-to-rump, compatible with approximately eight months of gestation. Gross findings included mild jaundice of the mucous membranes and carcass, moderate accumulation of light-yellow fluid within the thoracic cavity, and pulmonary collapse. The liver was markedly enlarged with diffuse moderate yellow-to-orange discoloration (Fig. 1A). Moderate splenomegaly was observed (Fig. 1B), and the kidneys showed diffuse moderate dark-red discoloration (Fig. 1C). Hepatic and perirenal lymph nodes were enlarged, with firm to smooth consistency and blackish-brown coloration (Fig. 1D). Multifocal hemorrhages were present on the parietal surface of the thymus. Blood smears were prepared during necropsy and stained with Panótico Rápido^®^ (Laborclin, Brazil) for Cytological evaluation. Tissue samples from the brain, skeletal muscle, eyelid, and major thoracic and abdominal organs were collected, fixed in 10% buffered formalin, routinely processed, and stained with hematoxylin and eosin for histological examination under light microscopy.

Genomic DNA was extracted from the spleen and from a pool of liver, spleen and lymph node samples using a commercial kit (PureLink Genomic DNA Mini Kit, Invitrogen, Carlsbad, CA, USA), following the manufacturer’s instructions, and stored at − 20 °C until analysis. DNA quantity and purity were assessed using a NanoDrop™ spectrophotometer (260/280 nm). PCR assays were performed according to the protocols described in Table 1. The pooled sample was subjected to PCR assays targeting *Leptospira* spp., Piroplasms, and *Anaplasma* spp., whereas the spleen sample was subjected to two distinct protocols: one specific for *B. bovis* and the other for *B. bigemina*. Amplified products were separated by electrophoresis on 1.5% agarose gels and visualized under UV transillumination. Amplicons of expected size were purified (PureLink kit, Invitrogen^®^) and sequenced using an automated Sanger sequencer. Generated sequences were compared with those available in GenBank using BLAST analysis. Additional blood, liver, spleen and lymph node samples were stored at − 20 °C for PCR testing for *Leptospira* spp., as previously described (Henker et al. 2020). Samples of fetal lung was sectioned at 3 μm, mounted on silanized slides (immunoslide/Easypath diagnostic slide Erviegas) and subjected to an IHC panel. Data on the antibodies used, dilution, antigen recovery and clone/brand used are found in Table 2.

**Table 1:** Primers used in the PCR protocol for the detection of Anaplasmataceae, Piroplasms, *Babesia bigemina*, *Babesia bovis* and *Leptospira* spp

Agent	Primers	Fragment size	Gene	Reference
Anaplasmataceae	F*: GE2 5’GTTAGTGGCATACGGGTGAAT 3’ R*: HE3 5’CTTCTATAGGTACCGTACATTATCTTCCCTAT 3’	360 bp	16 S rRNA	Breitschwerdt et al.,^ 1998^
Piroplasms	F: Bab2 F 5′ CCGTGCTAATTGTAGGGCTAATACA 3′ R: Bab2 R 5′ GCT TGA AAC ACT CTA RTT TTC TCA AAG 3′	≈ 500 bp	18 S rRNA	Soares et al. 2011
*Babesia bigemina*	F GAU5 5’TGGCGGCGTTTATTAGTTCG 3’ R GAU8 5’GCCAGCGAAAAGACCCAAC 3’	458 bp	18 S rRNA	Linhares et al., 2002
*Babesia bovis*	F GAU9 5’CTGTCGTACCGTTGGTTGAC 3’ R GAU10 5’ CGCACGGACGGAGACCGA 3’	541 bp	18 S rRNA	Linhares et al., 2002
*Leptospira* spp	F Lep 5’GGCGGCGCGTCTTAAACATG 3’ R Lep 5’TCCCCCCATTGAGCAAGATT 3’	330 bp	16 S rRNA	Merien et al., 1992

F* Forward, R* Reverse, *bp* base pairs

**Table 2:** Antibodies and antigen retrieval techniques used in immunohistochemistry to visualize leukocytes in lung tissue

Antibody	Dilution	Antigen retrieval	Clone/brand
MAC387	1:200	Citrato pH6.0	sc-66204/Santa Cruz
CD3	Ready-to-use	Citrato pH6.0	AB/Dako
CD20	1:200	Citrato pH6.0	L26/Dako

Cytological evaluation of blood smears revealed intraerythrocytic basophilic peripheral pinpoint structures measuring approximately 1 μm in diameter, consistent with *A. marginale* (Fig. 1E), as well as intraerythrocytic structures morphologically compatible with *Babesia*-like merozoites (Fig. 1F).

**Fig. 2:**
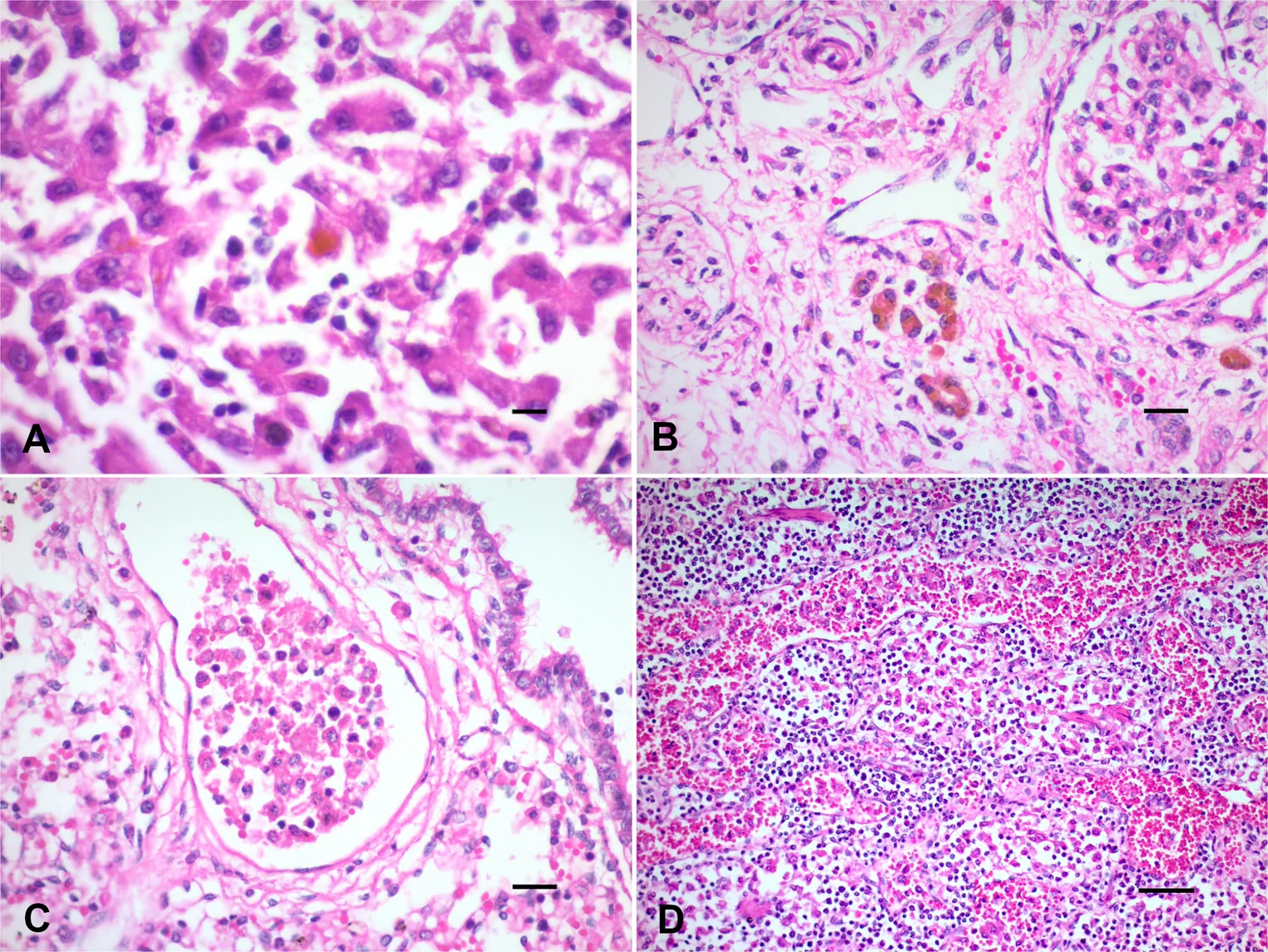
Microscopic findings of a bovine fetus with anaplasmosis and babesiosis**. A**. Liver. Marked bile accumulation is noted in the hepatocyte cytoplasm into biliary canaliculi. HE, obj. 63x, bar 10 µm. **B**. Kidney. A focal moderate accumulation of granular pigment in noted in the cytoplasm of epithelial tubular cells. HE, obj. 20x, bar 50 µm. **C**. Lung. Marked vascular congestion and prominent intravascular accumulation of inflammatory cells, predominantly mononuclear leukocytes, within pulmonary vessels (leukostasis). The alveolar septa are moderately thickened by cellular infiltration and edema, with reduced alveolar lumina. Multifocal intra-alveolar erythrocytes are also observed. HE, obj. 20x, bar 50 µm. **D**. Renal lymph node. Marked diffuse sinusoidal and vascular congestion, multifocal hemorrhage and moderate infiltration by mononuclear inflammatory cells, mainly macrophages and lymphocytes. Sinus expansion with increased cellularity and mild architectural disorganization are evident. HE, obj. 20x, bar 50 µm.

Microscopic lesions were most pronounced in the liver, kidneys, spleen, lymph nodes and lungs. The liver showed abundant golden-yellow pigment within bile ducts, hepatic sinusoids and hepatocytes (Fig. 2A). Renal lesions consisted of brownish pigment interpreted as hemoglobin casts within proximal tubular epithelial cells and tubular lumina (Fig. 2B). The spleen exhibited diffuse marked erythrophagocytosis associated with hemosiderosis. In the lungs there are macrophages with moderately abundant, occasionally vacuolated cytoplasm and are distributed within the sinuses and interstitium. Moderate amounts of brownish intra-alveolar material interspersed with amorphous eosinophilic structures morphologically compatible with meconium and keratin were observed. Leukocytostasis was present within vessels of the lungs (Fig. 2C), liver, kidneys, spleen, and lymph nodes. The lymph nodes there is marked vascular and sinusoidal congestion, with multiple distended vascular spaces filled with erythrocytes. There is expansion of the lymphatic sinuses by an abundant predominantly mononuclear cellular infiltrate composed of macrophages (Fig. 2D) and lymphocytes, associated with a moderate diffuse increase in parenchymal cellularity. Multifocal areas of hemorrhage and erythrocyte extravasation are noted, along with mild disruption of the normal cortical and medullary architecture.

PCR testing for *Leptospira s*pp. was negative. The pool sample tested positive for Piroplasms and *Anaplasma* spp. in the PCR. The spleen sample tested positive for *B. bovis* in the first-round cPCR using species-specific primers. Conversely, *B. bigemina* was only detected in a second-round PCR species-specific, utilizing the initial amplicons as a template alongside its species-specific primers (Table 1). The product of the PCR from the pool positive for *Anaplasma* spp. demonstrated 100% similarity with uncultured *Anaplasma* spp. sequences deposited in GenBank MG910977.1 from cattle. The sequence of this study was deposited under number: PZ437093.

**Fig. 3:**
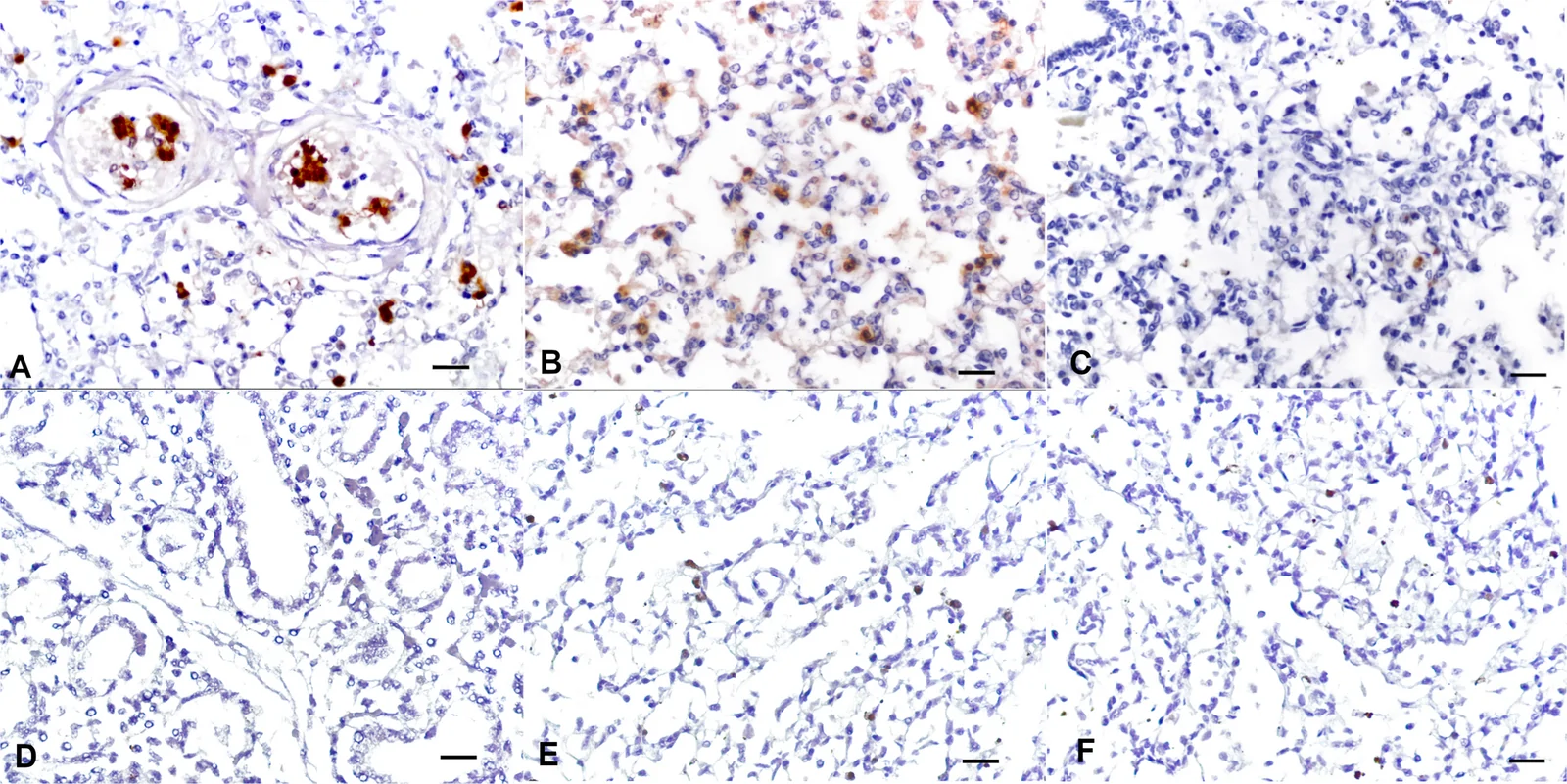
Immunohistochemical findings in the lung of a bovine fetus with hemolytic disease compared with a bovine fetus without lesions. **A**. Multifocal to moderate brown cytoplasmic immunolabeling is observed in perivascular and interstitial areas, highlighting a histiocytic infiltrate. IHC, MAC387, obj. 40x, bar 20 µm. **B**. Multifocal positive labeling of T lymphocytes is present, predominantly distributed along the alveolar septa and within the pulmonary interstitium. IHC, CD3, obj. 40x, bar 20 µm. **C**. Sparse to mild labeling of B lymphocytes is observed, with scattered distribution within the interstitium and in lower numbers compared to T lymphocytes. IHC, CD20, obj. 40x, bar 20 µm. **D**, **E** and **F**. Lung of a bovine fetus showing no staining for CD3, CD20 and MAC387

Immunohistochemistry demonstrated increase of macrophages (MAC387) (Fig. 3A), lymphocytes T (CD3) (Fig. 3B) and few lymphocytes B (CD20) (Fig. 3C) within vascular lumina of lungs, indicating intravascular accumulation of leukocytes.

Lung sections immunolabeled for MAC387 and CD3 were quantitatively evaluated and compared with control tissues from two bovine fetuses (five and nine months of gestation) obtained from a slaughterhouse, both lacking pulmonary lesions. For quantification, five images per immunolabel were captured using a 40× objective. Images underwent color deconvolution, and semi-automated counting of immunolabeled cells in alveolar walls was performed using regions of interest (ROI) in QuPath software (version 0.5.0).

In the fetus that tested positive via molecular and microscopic examination for *Anaplasma* spp (probably *(A) marginale*). and *(B) bovis* and *B. bigemina*, mean counts 36.8 MAC387-positive macrophages per field, and 27.6 CD3-positive per field (Table 3). No immunolabeling for MAC387 (Fig. 3D), CD3 (Fig. 3E) and CD20 (Fig. 3F) was detected in control animals.

**Table 3:** Summary of immunolabeled cell counts in the lung of a bovine fetus naturally infected with *Babesia* spp. and *Anaplasma* sp

*N*°	Immunohistochemistry	
	MAC387	CD3
1	33	19
2	33	29
3	24	27
4	53	39
5	41	24
Mean/Std	36.8 ± 10.87	27.6 ± 7.40
Total	184	138

This study was approved by the Animal Ethics Committee (Comissão de Ética no Uso de Animais – CEUA) of the Federal University of Mato Grosso (Protocol no. 23108.103506/2021-18).

## Discussion

Maternal infection by *A. marginale* can lead to progressive hemolytic anemia, which may culminate in fetal hypoxia, fetal death and abortion (Fowler and Swift 1975). Reduced oxygen delivery secondary to maternal anemia has been proposed as a major mechanism of fetal compromise, resulting from decreased fetal oxygen tension (pO₂) and increased carbon dioxide levels (pCO₂). Although the mechanisms involved in transplacental transmission of *A. marginale* remain incompletely understood, dissemination may occur during an extraerythrocytic phase or through the passage of infected erythrocytes across the placenta without significant pathogen amplification (Zaugg 1985; Aubry and Geale 2011). Additional factors, including immunosuppression, may favor subclinical maternal infection and increase the likelihood of vertical transmission (Silva and Fonseca 2014). In this case, the abortion was likely caused by fetal hemolytic anemia resulting from a co-infection with *Anaplasma* spp. (probably *(A) marginale*), *(B) bovis* and *B. bigemina*; since the cow showed no apparent clinical signs other than the abortion, she may have had a mild or subclinical infection, which allowed for vertical transmission.

The gross findings observed in this fetus including jaundice, splenomegaly and darkened kidneys are consistent with severe hemolytic disease and corroborate previous descriptions of fetal anaplasmosis and babesiosis (Fowler and Swift 1975; Henker et al. 2020). The identification of intraerythrocytic structures compatible with *A. marginale* and *Babesia* sp. in blood smears, together with PCR and sequencing results, provided strong etiologic evidence supporting co-infection as the cause of abortion. These findings reinforce the importance of integrating cytological, molecular and pathological data in abortion investigations, particularly in endemic regions where multiple hemoparasites circulate simultaneously.

Abortions and neonatal losses associated with anaplasmosis and babesiosis have been reported in different regions of Brazil (Costa et al. 2016; Henker et al. 2020; Andrade et al. 2024); however, the vascular leukocytostasis observed in the lungs, liver, kidneys, spleen and lymph nodes in the present case represents an unusual finding in bovine fetuses. This lesion may reflect an active fetal inflammatory response triggered by acute intrauterine infection. The intravascular accumulation of leukocytes observed in the pulmonary vessels may reflect local inflammatory responses triggered by hemoparasitic infection. Microvascular alterations and local hypoxia caused by erythrocyte destruction can promote endothelial activation and leukocyte adhesion, while parasite-derived and host inflammatory mediators may contribute to chemotactic recruitment and leukostasis. Together, these mechanisms suggest that the pulmonary lesions observed in this case are compatible with a systemic inflammatory process associated with intraerythrocytic infection (Adams and Lloyd 1997).

The fetus in this case was in the late stage of gestation, a period in which the bovine immune system reaches greater functional maturity. Fetal immune competence progressively develops throughout gestation, and phagocytic activity becomes more efficient in the final months due to maturation of cellular immune components and antigenic stimulation (Ahmed 2002). Experimental studies have demonstrated that bovine fetuses are capable of mounting cell-mediated immune responses, including IFN-γ production, during mid-to-late gestation, indicating progressive maturation of fetal immunocompetence (Andrianarivo et al. 2001; Rosbottom et al. 2008).

In agreement with this hypothesis, immunohistochemistry demonstrated prominent intravascular increase of MAC387-positive cells, indicating the participation of myeloid-lineage cells such as monocytes and macrophages. These findings suggest that the inflammatory response induced by *(A) marginale*. and *Babesia* sp. in the fetus may resemble mechanisms described in adult cattle, where acute infection by *(B) bovis* triggers strong cell-mediated immunity associated with erythrocyte destruction (Homer et al. 2000). The presence of CD3-positive further supports activation of the adaptive immune response, indicating that fetal immunity may play a more relevant role in hemoparasitic pathogenesis than previously recognized.

The predominance of intravascular monocyte–macrophage populations and marked leukostasis observed in the lungs may reflect a systemic fetal inflammatory response analogous to fetal inflammatory response syndrome (FIRS), which is characterized by systemic cytokine activation, multiorgan fetal inflammation, and adverse neonatal outcomes in human medicine (Jung et al. 2020).

The microscopic lesions observed including erythrophagocytosis, hemosiderosis, bilirubin accumulation and hemoglobin casts in renal tubules are consistent with severe hemolysis. Both intravascular and extravascular mechanisms likely contributed to erythrocyte destruction. Parasite replication inside erythrocytes and subsequent membrane rupture lead to intravascular hemolysis, while opsonization and macrophage-mediated phagocytosis contribute to extravascular hemolysis in the spleen and liver (Rimal et al. 2025). The resulting hemolytic process explains the observed jaundice, pigment accumulation and widespread macrophage activation.

Epidemiological factors may also have contributed to disease occurrence in this case. The Midwest region of Brazil is characterized by enzootic stability, where continuous exposure to ticks favors maintenance of hemoparasite circulation. Climatic instability and unseasonal rainfall recorded during the period preceding the abortion may have increased humidity and favored proliferation of *Rhipicephalus microplus* populations, consequently increasing infection pressure in the herd. Additionally, the genetic composition of the herd, which included animals with *Bos taurus taurus* ancestry, may have increased susceptibility to tick infestation compared with *Bos indicus* breeds, known for greater tick resistance (Bock et al. 1997; Brito et al. 2011). It is worth highlighting that the farm has a history of high calf mortality associated with this coinfection. Management factors, such as possible iatrogenic transmission through contaminated needles during oxytocin administration, should also be considered as potential contributors to pathogen dissemination (Kessler 2001).

Overall, this case highlights the multifactorial nature of bovine fetal losses associated with tick-borne pathogens. Environmental conditions, host susceptibility and pathogen burden likely interacted to enable transplacental infection and fetal disease. Importantly, the presence of marked intravascular leukocyte accumulation suggests that fetal immune responses may contribute significantly to lesion development, supporting the need for further studies investigating inflammatory pathways and immune activation in bovine fetuses affected by hemoparasitic infections.

## Conclusion

This report describes a case of bovine abortion associated with co-infection by *(A) marginale*,* (B) bovis* and *B. bigemina.*, confirmed by integrated cytological, molecular, and pathological findings. The gross and microscopic lesions were consistent with severe fetal hemolytic disease and the marked intravascular accumulation of leukocytes suggests that inflammatory responses may play an important role in lesion development and fetal death. Environmental conditions, host susceptibility and potential management-related factors likely contributed to disease occurrence in this endemic region. These findings highlight the importance of including tick-borne hemoparasites in differential diagnoses of bovine abortion and emphasize the need for further studies to better characterize fetal immune responses and inflammatory mechanisms associated with intrauterine hemoparasitic infections.

## Data Availability

No datasets were generated or analysed during the current study.
